# Regular Taekwondo Training Affects Mood State and Sociality but Not Cognitive Function among International Students in South Korea

**DOI:** 10.3390/healthcare9070820

**Published:** 2021-06-28

**Authors:** Ju-Yong Bae, Hee-Tae Roh

**Affiliations:** 1 Department of Physical Education, College of Arts and Physical Education, Dong-A University, Busan 49315, Korea; kosa99@dau.ac.kr; 2 Department of Sports Science, College of Health Science, Sun Moon University, Asan-si 31460, Korea

**Keywords:** physical activity, physical fitness, mental health, sociality, cognition, international students

## Abstract

We aimed to investigate the effect of Taekwondo training on physical fitness, mood, sociality, and cognitive function among international students in South Korea. We randomly assigned 24 international students to a control group (CG, *n* = 12) and experimental group (EG, *n* = 12). The EG performed Taekwondo training for 16 weeks, while the CG did not train. Each participant underwent a physical fitness test and sociability questionnaire before and after the intervention. We also examined changes in mood state and cognitive function, using the Korean version of the Profile of Mood State-Brief (K-POMS-B), and the Stroop Color and Word test, respectively. Regarding the physical fitness variables, sit-and-reach records in the EG significantly increased after intervention (*p* < 0.05). In the sub-variable of K-POMS-B, Vigor-Activity scores significantly increased (*p* < 0.05) after intervention, while the Fatigue-Inertia scores significantly decreased in the EG (*p* < 0.05). Furthermore, in the EG, peer relationship scores, a sub-variable of sociability, significantly decreased after intervention (*p* < 0.05). These findings suggest that Taekwondo training can not only improve flexibility among physical fitness factors, but can also be effective in improving the mood state and sociality of international students.

## 1. Introduction

Attracting and hosting international students are a means of acquiring and retaining an excellent workforce that can influence future national competitiveness [1]. This effect is not limited to the development of individual international students, but also greatly affects the international competitiveness of the host countries. The Korean government has set a target of attracting 200,000 excellent international students by 2023, to counteract the recent trend of a deficit in the balance of international students, a sharp decline in the school-age population, and a decrease in the working-age population. The number of international students in Korea increased more than 10 times, from 12,314 in 2003 to 160,165 in 2019 [1]. In terms of nationality, as of 2019, 44.4% of all international students studying in South Korea were from China and 23.4% from Vietnam, indicating a high proportion of Asian students [1].

In general, international students experience more difficulties in adjusting to college life in the host country than they would have experienced in their home country. It has been reported that during this adaptation period, international students are under considerable stress [2,3]. Language barriers not only affect academic performance, but also interfere with interaction and communication with classmates, and, thus, are a major stressor for international students [4]. Additionally, transcultural difficulties, such as religious differences, can also act as a stressor for international students [5]. Further, homesickness, unfamiliar foods, health and financial issues, future career plans, everyday life, and differences in the education systems are major problems that these international students face [3,5]. Such adaptation related stress involves specific stress behaviors in psychological and social aspects, accompanied by physical symptoms [6]. International students in South Korea are no exception. Although the number of international students in South Korea is increasing, they experience poor physical, psychological, and social well-being, given to the problems in academic performance due to language barriers and difficulties in adapting to the new environment, making it difficult for them to successfully complete their degree [7,8].

Taekwondo, a traditional Korean martial art, has emerged as a popular sport enjoyed by more than 80 million people, regardless of age and gender, in more than 180 countries around the world [9,10]. Previous research indicates that Taekwondo training has a positive impact on physical health, such as improving physical fitness and body composition, and also on psychological, social, and cognitive health [9,10,11]. Specifically, Kim et al. reported that 12 weeks of Taekwondo training can induce beneficial changes in skeletal muscle fitness, flexibility, and the body composition of adolescent females [11]. Furthermore, Fong and Ng reported that regular Taekwondo training improves aerobic fitness and flexibility, highlighting its benefits [9]. In addition, Roh et al. reported that 16 weeks of Taekwondo training was effective in improving the mood and sociality of children from multicultural families in South Korea [10]. Another previous study on male undergraduate students indicated that after 8 weeks of Taekwondo training, scores on the Stroop test, which evaluates cognitive function, significantly increased [12]. Additionally, undergraduate students may suffer from lower levels of psychological and/or mental well-being than in the general population, including in same aged non-student populations, due to multiple stress factors, such as academic challenges, competition, and pressure to achieve [13,14,15]. Taekwondo training can be effective in improving the well-being of undergraduate students because it has a beneficial effect on stress management [16], as shown by Toskovic who proved that Taekwondo can be effective in improving the mood state of undergraduate students [17]. Furthermore, it has been reported that Taekwondo participation can contribute to personal growth related to cross-cultural contacts. This has been shown through providing rich opportunities to promote cultural and ethnic understanding related to East Asian culture and social interactions with other ethnic groups [18].

The assessment of successful adaptation to university life in international students should take into account both academic achievement and aspects of healthy growth and development, including physical and mental health. When compared to problems of language barriers and academic performance, addressing health problems can contribute more toward improving students’ adaptation and life satisfaction in the host country [8]. However, despite international students in Korea experiencing a deterioration in health from various causes, the policies related to welfare, psychosocial environment, and health care for international students by the government and universities of South Korea have not been adequate [3,8]. Therefore, the aim of this study was to examine the effects of regular Taekwondo intervention on physical fitness, mood, sociality, and cognitive function for international students studying in South Korean universities. This was carried out from a holistic perspective of health promotion.

## 2. Methods

### 2.1. Participants

The prospective number of participants was calculated to be 24, for an effect size of 0.40 at α value of 0.05 and a desired statistical power (1-β) of 0.95. We selected 24 healthy international students who were able to communicate in Korean and were enrolled in undergraduate programs at Korean universities. The international students’ nationalities were Chinese and Vietnamese. Participants were randomly assigned to the control group (CG, *n* = 12) or the experimental group (EG, *n* = 12). The EG participated in Taekwondo training for 16 weeks, and the CG participated in liberal arts classes offered at the universities as well as sports activities, such as bowling, table tennis, and golf, during the same period. The study protocol was approved by the institutional review board of Dong-A University (IRB No. HR-035-08) and conformed to the standards set by the latest revision of the Declaration of Helsinki. All participants were fully informed of the study procedures and signed an informed consent form, indicating that they understood the study procedures and the risks and benefits of participation. It was also indicated that withdrawal from participation would confer no negative consequences. The characteristics of the participants at baseline are shown in Table 1. There were no significant differences between the groups.

### 2.2. Research Procedure

Study procedures included participant selection, physique measurement, pre-test, and post-test. Testing was conducted before (pre-) and after (post-) 16 weeks of intervention, by selecting indices that assessed the physical fitness, mood state, sociability, and cognitive function of each participant. Measurement variables were selected according to previously established methodology [10]. Specifically, physique (height, weight, body mass index, and percentage of body fat) was measured using a multi-frequency bioelectrical impedance analyzer (Accuniq BC720; SELVAS Healthcare, Daejeon, Korea). Physical fitness was measured by cardiorespiratory endurance (maximal oxygen uptake (VO_2_max)), strength (grip strength and back strength), flexibility (sit-and-reach), power (Sargent jump), and balance (Stork test). Specifically, cardiorespiratory endurance was measured by estimating VO_2_max with the Ebbeling protocol on a treadmill (T150; Cosmed, Rome, Italy), and by wearing wireless heart rate measuring equipment (Polar-a5; Polar, Kempele, Finland) [19]. Grip and back strength were measured twice in 0.1 kg units, using digital grip (GRIP-D; Takei, Niigata, Japan) and back strength (BACK-D; Takei, Niigata, Japan) measuring equipment. The highest score was recorded for analysis. Sit-and-reach, Sargent jump, and Stork test were measured twice using basic fitness measuring equipment (Helmas-III; O2run, Seoul, Korea), and the best scores were recorded for analysis.

The mood state was estimated using the Korean Version of the Profile of Mood State-Brief (K-POMS-B) by Yeun and Shin-Park, who verified its reliability and validity and adapted it for Korea from the profile of mood states (POMS) originally by McNair et al. [20,21]. This questionnaire consists of a total of 30 items, which are rated on a 5-point Likert scale (0 = not at all, 1 = a little, 2 = moderately, 3 = quite a bit, and 4 = extremely), and is divided into six sub-areas: Tension-Anxiety, Depression-Dejection, Anger-Hostility, Vigor-Activity, Fatigue-Inertia, and Confusion-Bewilderment.

The sociability measuring model for international students by Kim was used to evaluate sociability [22]. It consists of 13 items: peer relationship (6 items), social activity (3 items), and perceived discrimination (4 items). In this sociability measurement model, a lower sub-area score indicates higher sociability, and a higher sub-area score indicates lower sociability, excluding the social activity items.

Cognitive function was measured using the Stroop Color and Word test (Catalog No. 30150M) developed by Golden and Freshwater [23]. The Stroop Color and Word test consists of three conditions: word reading (Word), color reading (Color), and color-word reading (Color-Word). Each condition consists of 100 items per page with 5 columns and 20 rows. The participants were asked to read the words aloud within 45 s, and to read as many as possible under each condition (Word, Color, and Color-Word). In this study, the participants’ raw scores of words correctly read aloud for each condition within the allocated time limit were recorded. The higher the recorded score, the better the cognitive function.

### 2.3. Taekwondo Training Intervention

The Taekwondo training consisted of a training session lasting 60 min: 5 min of warm-up and cool-down, and 50 min of main exercise. Sessions were performed once a week at 50–80% maximal heart rate (HRmax), for 16 weeks. All interventions were conducted by a Taekwondo expert instructor through demonstration and coaching, and the main exercise consisted of 10 min of basic fitness, such as shuttle runs, the Burpee test, and leapfrog. Then there was 5 min of 6 basic Taekwondo motions (close stance, parallel stance, riding stance, forward stance, forward inflection stance, and backward inflection stance) and body punching in horse-riding stance. This was followed by 10 min of Poomsae from the 1st to 8th chapters, 10 min of kicking sessions with basic kicking (front kick, turning kick, side kick, and downward kick) and steps as well as mitt kicks. Finally, there was 15 min of Taegeuk gymnastics.

### 2.4. Statistical Analyses

An independent *t*-test and paired *t*-tests were conducted for statistical analysis. Statistical analyses were performed with SPSS version 25.0 for Windows (SPSS Inc., Chicago, IL, USA). Data are presented as the mean ± standard deviation (SD). A two-way repeated measured analysis of variance (ANOVA) was conducted to examine the differences between the groups in each dependent variable. Tests of normality for all measured values were conducted using the one-sample Kolmogorov–Smirnov significant interaction, and the level of statistical significance was set at 0.05.

## 3. Results

### 3.1. Changes in Physical Fitness According to Taekwondo Intervention

Changes in physical fitness in the CG and EG pre- and post-intervention are shown in Table 2. The two-way repeated measured ANOVA on physical fitness revealed a significant difference in sit-and-reach (*F* = 25.574, *p* < 0.001). Post-hoc analysis indicated no significant difference between pre- and post-intervention in the CG. Alternatively, in the EG, sit-and-reach scores significantly increased post- compared to pre-intervention (*p* < 0.05). However, there was no significant difference in VO_2_max (*F* = 1.101, *p* = 0.305), grip strength (*F* = 0.984, *p* = 0.332), back strength (*F* = 0.971, *p* = 0.335), Sargent jump (*F* = 0.854, *p* = 0.365), or Stork test (*F* = 0.014, *p* = 0.906).

### 3.2. Changes in Mood State According to Taekwondo Intervention

Changes in mood state in the CG and EG pre- and post-intervention are shown in Table 3. The two-way repeated measures ANOVA on mood state revealed a significant difference in Vigor-Activity (*F* = 21.359, *p* < 0.001) and Fatigue-Inertia (*F* = 6.600, *p* = 0.018). Post-hoc analysis indicated no significant difference between pre- and post-intervention among the CG. Alternatively, in the EG, Vigor-Activity scores significantly increased post- compared to pre-intervention (*p* < 0.05), and Fatigue-Inertia scores significantly decreased post- compared to pre-intervention (*p* < 0.05). However, there was no significant difference in Tension-Anxiety (*F* = 0.116, *p* = 0.737), Depression-Dejection (*F* = 2.902, *p* = 0.103), Anger-Hostility (*F* = 0.478, *p* = 0.496), or Confusion-Bewilderment (*F* = 0.227, *p* = 0.639).

### 3.3. Changes in Sociability According to Taekwondo Intervention

Changes in sociability in the CG and EG pre- and post-intervention are shown in Table 4. The two-way repeated measures ANOVA on sociability revealed a significant difference in peer relationship (*F* = 25.574, *p* < 0.001). Post-hoc analysis indicated no significant difference in the CG between pre- and post-intervention. Alternatively, in the EG, peer relationship scores decreased significantly post- compared to pre-intervention (*p* < 0.05). However, there was no significant difference in social activity (*F* = 0.008, *p* = 0.931) or perceived discrimination (*F* = 0.328, *p* = 0.573).

### 3.4. Changes in Cognitive Function According to Taekwondo Intervention

Changes in cognitive function in the CG and EG pre- and post- intervention are shown in Table 5. The two-way repeated measures ANOVA on cognitive function indicated no significant difference in scores for Word (*F* = 0.743, *p* = 0.398), Color (*F* = 0.000, *p* = 0.984), or Color-Word (*F* = 0.050, *p* = 0.825).

## 4. Discussion

Participation in routine Taekwondo training can provide adolescents with various physical, mental, and social benefits [24], and can be beneficial in the stress management of undergraduate students [16]. In addition, it can be effective in promoting holistic health, by inducing positive changes in body, mind, and spirit [16]. Further, Taekwondo has inherent Korean characteristics, such as the specificity of terms and techniques written in Korean, and the norms of etiquettes, reflecting Korean perspectives; thus, it has the ability of providing an experience of the unique culture of Korea [25]. International students seek a variety of experiences in Korean culture to understand and adapt to the culture of South Korean society. Therefore, learning Korean culture through physical activities can be effective [26]. Ahn et al. reported that through participation in Taekwondo, foreigners have opportunities to learn the spiritual values that Koreans cherish, as well as make friends, and experience a cultural exchange [27]. Kim reported that Taekwondo is a sport that is familiar to international students, even with little knowledge and understanding of Korean culture. Thus, experiencing Taekwondo would benefit international students in adapting to South Korean society [25].

In this study, physical fitness was assessed to verify the effects of Taekwondo training on the physical health of international students. The results indicated no significant differences in VO_2_max, grip strength, back strength, Sargent jump, or Stork test scores, which are indicators for cardiorespiratory fitness, muscular strength, power, and balance, respectively. These results may be due to the failure to provide sufficient exercise in this study to induce changes in physical fitness. The American College of Sports Medicine recommends an exercise frequency in healthy participants of 3–5 times weekly, with 3 times weekly being the minimum exercise frequency required for improvement in physical fitness, such as cardiorespiratory fitness, muscular strength, muscular endurance, and power [28]. However, in this study, Taekwondo intervention was performed only once a week, and previous studies [29,30] that reported improvement of physical fitness through Taekwondo training performed the intervention three times or more weekly. Alternatively, in this study, the sit-and-reach score, an indicator of flexibility, increased significantly after intervention, although the frequency of Taekwondo training remained at once weekly. This result indicates that Taekwondo can improve flexibility, and supports previous studies reporting improvement in flexibility with exercise training only once a week. For example, Tolnai et al. conducted Pilates exercise intervention once weekly for 10 weeks for young, sedentary women, and the sit-and-reach scores increased significantly [31]. Furthermore, Grabara and Szopa reported a significant increase in the range of motion as a result of weekly yoga exercise for 20 weeks among women aged 50 years or older [32]. In addition, Mayorga et al. conducted an intervention using a physical education-based stretching program for 180 high school students over eight weeks, in which groups were divided by frequency into once and twice a week. The sit-and-reach scores were measured, and they increased significantly with just once weekly exercise, with no additional effect of improvement in hamstring extensibility with twice-weekly exercise [33].

Participation in exercises, including regular physical activities, can induce positive changes in mental and social health, as well as improvement in physical fitness [34,35]. Vankim and Nelson reported that physical activities should be promoted to improve college students’ mental well-being [34], and Garcia et al. reported a close association between physical activities and sociability [35]. Participation in Taekwondo training can also be effective in emotional and social development, through reducing anxiety, promoting independence and leadership, and controlling aggression [36,37]. In addition, some previous studies have suggested that regular Taekwondo training can be effective in improving mood states, as measured by the POMS test [10,38]. In this study, to verify the effect of Taekwondo training on international students’ mental and social health, a K-POMS-B test and a sociality survey, consisting of three sub-categories of peer relationships, social activity, and perceived discrimination, were conducted. The results indicated that among the sub-categories of K-POMS-B, the Vigor-Activity score increased significantly, and the Fatigue-Inertia score decreased significantly. In addition, among the sub-categories of the sociality questionnaire, the peer relationship score decreased significantly after Taekwondo training. These results indicate that Taekwondo training can effectively improve international students’ mental and social health, and support the findings of previous studies [10,17,39], that reported an improvement in mood state and sociality through Taekwondo training participation. In a previous study by Toskovic, 40 college-age students were assigned to the Taekwondo group and the control group, with 20 students in each group. Scores for the Vigor and Fatigue items of the POMS test in the Taekwondo group improved significantly [17]. Furthermore, Trulson reported that six months of Taekwondo training may promote psychosocial health more than modern martial arts training. This finding is suggested to be due to the nature of Taekwondo, that places greater value on respect, humility, responsibility, perseverance, and honor, compared to other martial arts [39].

Taekwondo training is an exercise program with beneficial effects for improving cognitive function, and is recommended as a school physical education class [40]. Kim demonstrated a mechanism for improving the cognitive function of undergraduate students through Taekwondo training [12]. In this study, the Stroop Color and Word test was conducted to verify the effects of Taekwondo training on the cognitive health of international students, but no significant difference in any of the Word, Color, or Color-Word scores was observed. This may be due to the low exercise frequency, similar to the lack of differences observed in physical fitness results in this study. Kim reported a significant improvement in cognitive function after Taekwondo training intervention 5 times weekly for 8 weeks [12]. Additionally, previous studies verifying the effect of Taekwondo training on cognitive function among students also support the findings of this study [10,29]. Specifically, as a result of Taekwondo training intervention 5 times weekly for 16 weeks, an improvement in cognitive function was indicated by the significant increase in Color-Word test scores [29]. However, with Taekwondo training at a once weekly frequency, the Stroop Color and Word test scores did not change significantly [10].

The important findings from these results are that Taekwondo training produces an improvement in holistic health for international students who may experience poor physical, psychological, and social health due to difficulties in adapting to the host country. However, this study has limitations. Firstly, the results of this study should be generalized with caution because of the nationality of the international students participating in this study was limited to Chinese and Vietnamese. Furthermore, it was not possible to verify the Korean language ability of the international students. In addition, there is a possibility that there was no significant change in variables due to an insufficient frequency of exercise, which was once weekly in this study. It is unclear through what mechanism Taekwondo had an effect on the improvement of mood state and sociality. Therefore, in future studies, further verification is required by adjusting the frequency of exercise, to ensure the effect of exercise for international students of more diverse nationalities. In addition, it is considered necessary that a study prove the specific effect of Taekwondo through comparative verification with a specific exercise type (sport).

## 5. Conclusions

In conclusion, a regular routine of Taekwondo training did not induce changes in physical fitness, such as cardiorespiratory fitness, muscular strength, power, and balance, and in cognitive functions of international students. However, the training was effective in improving mood and sociability, as well as flexibility. The results of this study indicate that Taekwondo intervention may benefit the health of international students studying in South Korean universities.

## Figures and Tables

**Table 1:** The characteristics of the participants at baseline.

Variable	CG (*n* = 12)	EG (*n* = 12)	*p*
Gender (male/female)	7/5	7/5	
Age (years)	23.25 ± 4.31	22.42 ± 4.40	0.644
Height (cm)	163.44 ± 7.23	165.73 ± 5.31	0.387
Weight (kg)	58.23 ± 9.26	57.60 ± 8.09	0.860
BMI (kg/m^2^)	21.73 ± 2.49	20.97 ± 2.67	0.493
Body fat (%)	23.50 ± 4.23	21.21 ± 6.51	0.318

Values are presented as mean ± SD. CG, control group; EG, experimental group; BMI, body mass index. *p* value was determined using the independent t-test for each of the two groups at baseline.

**Table 2:** Changes in physical fitness.

Variable	CG (*n* = 12)		EG (*n* = 12)		Interaction (Group × Time)	
	Pre	Post	Pre	Post	*F*	*p*
VO_2_max (ml/kg/min)	47.95 ± 3.10	48.49 ± 3.10	48.42 ± 4.82	48.18 ± 4.25	1.101	0.305
Grip strength (kg)	36.68 ± 12.76	36.00 ± 13.03	34.17 ± 10.32	34.28 ± 11.16	0.984	0.332
Back strength (kg)	86.88 ± 42.10	86.33 ± 41.37	83.54 ± 31.77	83.75 ± 32.20	0.971	0.335
Sit-and-reach (cm)	13.02 ± 9.37	12.86 ± 9.56	10.99 ± 7.65	13.10 ± 7.46 ^#^	25.574	< 0.001 ***
Sargent jump (cm)	34.67 ± 7.09	34.33 ± 7.23	38.25 ± 8.60	37.25 ± 8.64	0.854	0.365
Stork test (sec)	34.75 ± 29.25	37.00 ± 31.10	36.75 ± 23.51	39.83 ± 24.04	0.014	0.906

Values are presented as mean ± SD. CG, control group; EG, experimental group; VO_2_max, maximal oxygen uptake. ^#^ Compared with baseline values within the EG (*p* < 0.05); *** *p* < 0.001.

**Table 3:** Changes in mood state.

Variable	CG (*n* = 12)		EG (*n* = 12)		Interaction (Group × Time)	
	Pre	Post	Pre	Post	*F*	*p*
Tension- Anxiety (score)	1.73 ± 0.61	1.70 ± 0.47	1.75 ± 0.53	1.75 ± 0.43	0.116	0.737
Depression- Dejection (score)	1.27 ± 0.49	1.33 ± 0.45	1.43 ± 0.42	1.35 ± 0.44	2.902	0.103
Anger- Hostility (score)	1.38 ± 0.45	1.35 ± 0.34	1.53 ± 0.51	1.43 ± 0.47	0.478	0.496
Vigor- Activity (score)	2.58 ± 0.34	2.60 ± 0.30	2.52 ± 0.49	2.87 ± 0.41 ^#^	21.359	< 0.000 ***
Fatigue- Inertia (score)	1.95 ± 0.85	1.95 ± 0.74	2.05 ± 0.55	1.65 ± 0.35 ^#^	6.600	0.018 *
Confusion- Bewilderment (score)	1.68 ± 0.67	1.58 ± 0.52	1.75 ± 0.39	1.72 ± 0.43	0.227	0.639

Values are presented as mean ± SD. CG, control group; EG, experimental group. ^#^ Compared with baseline values within EG (*p* < 0.05); *** *p* < 0.001; * *p* < 0.05.

**Table 4:** Changes in sociability.

Variable	CG (*n* = 12)		EG (*n* = 12)		Interaction (Group × Time)	
	Pre	Post	Pre	Post	*F*	*p*
Peer relationship (score)	2.63 ± 0.52	2.75 ± 0.44	2.96 ± 0.38	2.56 ± 0.32 ^#^	13.665	0.001 **
Social activity (score)	1.90 ± 0.63	1.81 ± 0.53	2.05 ± 0.76	1.98 ± 0.62	0.008	0.931
Perceived discrimination (score)	1.80 ± 0.30	1.78 ± 0.26	1.83 ± 0.25	1.73 ± 0.27	0.328	0.573

Values are presented as mean ± SD. CG, control group; EG, experimental group. ^#^ Compared with baseline values within EG (*p* < 0.05); ** *p* < 0.01.

**Table 5:** Changes in cognitive function.

Variable	CG (*n* = 12)		EG (*n* = 12)		Interaction (Group × Time)	
	Pre	Post	Pre	Post	*F*	*p*
Word (score)	92.25 ± 15.15	95.33 ± 9.59	92.33 ± 18.27	93.00 ± 14.50	0.743	0.398
Color (score)	70.83 ± 15.87	69.50 ± 10.66	68.50 ± 23.18	67.08 ± 16.33	0.000	0.984
Color- Word (score)	50.25 ± 16.65	50.42 ± 11.00	49.67 ± 13.03	50.58 ± 11.28	0.050	0.825

Values are presented as mean ± SD. CG, control group; EG, experimental group.

## Data Availability

Data generated and analyzed during this study are included in this article. Additional data are available from the corresponding author on request.
